# Continuous paravertebral block using a thoracoscopic catheter-insertion technique for postoperative pain after thoracotomy: a retrospective case-control study

**DOI:** 10.1186/s13019-017-0566-8

**Published:** 2017-01-25

**Authors:** Yoshikane Yamauchi, Mitsuhiro Isaka, Kamon Ando, Keita Mori, Hideaki Kojima, Tomohiro Maniwa, Shoji Takahashi, Eiji Ando, Yasuhisa Ohde

**Affiliations:** 1 Division of General Thoracic Surgery, Shizuoka Cancer Center, 1007 Shimonagakubo, Nagaizumi, Shizuoka 411-8777 Japan; 2 Division of Anesthesiology, Shizuoka Cancer Center, Shizuoka, Japan; 3 Clinical Trial Coordination Office, Shizuoka Cancer Center, Shizuoka, Japan

**Keywords:** Paravertebral block, Thoracotomy, Postoperative pain

## Abstract

**Background:**

Thoracic epidural analgesia (EDA) is the gold standard for pain control after thoracotomy. However, because of its severe side effects, it is contraindicated in patients taking anticoagulant or antiplatelet drugs. In addition, some patients’ anatomy can make epidural catheter insertion challenging. We therefore investigated the safety and efficacy of paravertebral block (PVB) using a thoracoscopic insertion technique, which avoids damage to the parietal pleura, for postoperative pain after thoracotomy.

**Methods:**

Patients who underwent thoracotomy with thoracic PVB in our hospital between March 2013 and March 2014 were examined retrospectively. Prior to creating the thoracotomy incision, a catheter for PVB was inserted percutaneously into the paravertebral space under thoracoscopic guidance. A matched-pair control group was selected at a 1:2 ratio from patients who underwent thoracotomy with thoracic EDA in our hospital from April 2011 to February 2013. Pain control and side effects were compared between groups and the results statistically analyzed.

**Results:**

Thoracic PVB was performed in 56 patients during this period, and 112 patients were selected as matched controls. Numeric Rating Scale scores on postoperative day 2 did not differ significantly between the PVB group (3.25 ± 1.80) and the EDA group (3.56 ± 2.05) (*p* = 0.334). In terms of side effects, urinary retention occurred less frequently in thoracic PVB patients (*p* = 0.03).

**Conclusion:**

Under the conditions of the present study, continuous thoracic PVB was at least as effective as epidural analgesia for postoperative pain control after thoracotomy with lung resection.

**Electronic supplementary material:**

The online version of this article (doi:10.1186/s13019-017-0566-8) contains supplementary material, which is available to authorized users.

## Background

Post-thoracotomy pain is considered one of the most intense types of postoperative pain because a thoracotomy incision involves multiple muscle layers and possible rib injury. Effective treatment of acute post-thoracotomy pain after lung resection is particularly important to keep the patient comfortable and to minimize pulmonary complications [1, 2]. Thoracic epidural analgesia (EDA) is the gold standard for pain control after thoracotomy. However, because of its severe side effects, it is contraindicated in patients taking anticoagulant or antiplatelet drugs, which have recently become more frequently used. In addition, some patients’ anatomy can make epidural catheter insertion challenging. Therefore, it is important to develop an alternative procedure.

Thoracic paravertebral block (PVB) blocks the nerves of multiple contiguous thoracic dermatomes above and below the infusion site [3] and has been shown to provide pain relief comparable to that of EDA but with fewer side effects [4, 5]. In addition, a paravertebral catheter can easily be inserted intraoperatively by the surgeon under direct vision, eliminating the need for a separate procedure.

The efficacy of thoracic PVB depends on accurate catheter placement and on the potency, concentration, and volume of local anesthetic. Our preliminary trials of thoracic PVB led us to suspect a strong correlation between a number of reported insertion methods [3, 4, 6–10] and substantial leak of local anesthetic into the pleural space, which results in less effective postoperative pain control. However, some authors have described the effectiveness of intraoperative percutaneous catheter placement during video-assisted thoracoscopic surgery (VATS) [11, 12]. This insertion method offers better pain control, as the extra-pleural space is closed above the catheter tip, allowing no leakage into the pleural space and facilitating easy coverage of more than one intercostal space. We applied this method because of its potential usefulness, not only in VATS, but also when used just before a thoracotomy incision.

At our institution, we have performed thoracic PVB using this insertion technique since March 2013, applying it to thoracotomy patients determined by the anesthesiologist to be contraindicated for EDA. In this study, using sample matching, we retrospectively analyzed the relationship between analgesic technique and pain-control outcome in thoracotomy patients.

## Methods

### Ethics, consent and permissions

The Shizuoka Cancer Center Hospital Institutional Review Board approved the retrospective collection and analysis of data from medical records of patients included in this study (approval ID: 25-J128-25-1-3). The need for informed consent from each patient was waived.

### Patients

Patients who underwent thoracotomy for lung resection at our institution between March 2013 and March 2014 were examined retrospectively. Cases were discussed by anesthesiologists and surgeons, and those patients determined to be contraindicated for EDA, or whose anatomy would make epidural catheter insertion challenging, were selected for thoracic PVB.

### Thoracotomy procedure

General anesthesia was induced with 1.5–2 mg/kg of propofol, 2 μg/kg of fentanyl, and 0.6 mg/kg of rocuronium and maintained with volatile anesthesia or total intravenous anesthesia. All patients were intubated with a double-lumen endobronchial tube and ventilated mechanically.

Patients were placed in the lateral decubitus position. Before the thoracotomy was performed, thoracoscopic intrathoracic inspection was conducted in each patient as follows: First, a 5-mm port for the camera was placed in the 7th intercostal space at the midaxillary line. After confirming that there was no evidence of malignant pleural effusion or pleural dissemination, a thoracotomy was created in the 4th or 5th intercostal space using a posterolateral or anterior axillary approach. Upon completion of the thoracotomy, a chest tube was placed in the 7th intercostal space through the camera port. Thoracotomy and wound closure were accomplished in the same manner in the PVB and EDA groups.

Patients were begun on a regimen of oral COX-2 inhibitor and pregabalin. The chest tube was removed if there was no leakage and the pleural effusion amounted to less than 200 mL daily. Other protocols of postoperative management were also the same in both groups.

### PVB procedure

After confirmation that there was no dissemination, but before initiation of the thoracotomy, the paravertebral catheter was inserted in a manner that has been previously described [11–14], as follows: The upper edge of the spinous process of the T5 vertebral body was palpated through the skin. With the assistance of forceps inserted through the same camera port, the paravertebral space was visualized under thoracoscopy. An 18-gauge Tuohy epidural needle was inserted at a point 3 cm lateral to the lateral edge of the vertebra. The Tuohy needle was carefully advanced, without puncturing the parietal pleura, until it reached the paravertebral space over the superior border of the transverse process, where 20 mL of normal saline was injected to expand the paravertebral space. Next, while holding the Tuohy needle steady, the paravertebral catheter was placed through the needle and the needle was removed, making sure that the tip of the catheter remained in place. Advancement of the needle and entry of catheter into the paravertebral space were continuously monitored under thoracoscopic vision. To ensure proper positioning of the catheter tip, 20 mL of 0.375% ropivacaine was injected through the catheter. Correct placement was indicated by expansion of the extrapleural space without leakage of local anesthetic into the pleural space (Video 1). The catheter was secured with 2-0 nylon sutures, and continuous infusion of 0.45% ropivacaine was started as soon as possible. The rate of infusion was initially 6–8 mL/h but was titrated to patient comfort. In addition, intravenous fentanyl 20–40 μg/h was continued was continued for the remainder of the day of surgery.



**Additional file 1: Video 1.** PVB procedure in the case of right lower lobectomy. The catheter was placed in the sixth intercostal space, followed by the fifth intercostal space of thoracotomy. (MOV 147456 kb)


### Selection of the control group

The matched-pair control group (EDA group) was selected on a 1:2 ratio from patients who underwent thoracotomy with EDA in our hospital from April 2011 to February 2013. Thoracic epidural catheters were inserted before the induction of general anesthesia at the level of T6-7 and secured in place. EDA was also used for intraoperative analgesia. Patients received a mixture of 0.2% ropivacaine with fentanyl as local anesthetic. The initial dose was 5 mL/h, but this was titrated to patient comfort.

Matching criteria were sex, age, and type of surgery. The criterion of age was divided into five groups: ≤49, 50–59, 60–69, 70–79, and 80–89 years. The types of surgery were divided into three groups: lobectomy with mediastinal lymph node dissection, lobectomy without mediastinal lymph node dissection, and all others. The control group was selected by a person not otherwise associated with the study with no other information about the patients. When there were more than three matching controls for a PVB patient, we selected patients using a random-number table.

### Statistical analysis

All relevant patient data were recorded before and after surgery, and patients were followed until hospital discharge. The following data were assessed: 1) pain score 48 h after surgery, 2) requirement for intravenous rescue analgesia, 3) the required duration of regional anesthesia, and 4) the amount of fentanyl or ropivacaine administered during the perioperative period.

Categorical variables were compared using Fisher’s exact test and continuous variables using the Mann-Whitney test. IBM SPSS for Windows, Version 22.0 (IBM Corp., Armonk, NY, USA) was used for all statistical evaluations. *P* values less than 0.05 were considered statistically significant.

## Results

Between March 2013 and February 2014, 244 patients underwent thoracotomy at our institution. Of these, 56 were contraindicated for EDA and therefore underwent the thoracic PVB procedure (PVB group). Contraindications were anti-platelet treatment (31 cases, 55.4%), therapeutic anticoagulation (nine cases, 16.0%), hemostatic disorders and/or coagulopathies (four cases, 7.1%), and potentially technically difficult epidural catheter insertion (12 cases, 21.4%). The types of surgery performed in this group were lobectomy (38 cases, 67.9%), segmentectomy (10 cases, 17.8%), wedge resection (seven cases, 12.5%), and thymectomy (one case, 1.8%).

**Fig. 1:**
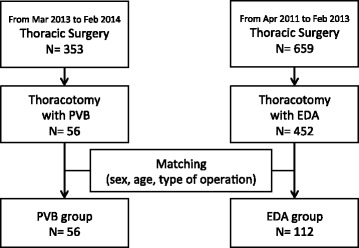
Flowchart of patient enrollment. EDA, epidural analgesia; PVB, paravertebral block

The matched-control group (EDA group) was selected from 452 patients who received thoracotomy with EDA at our hospital from April 2011 to February 2013. After matching, this group consisted of 112 patients (Fig. 1).

The characteristics of the two groups are shown in Table 1. The PVB group had a tendency toward higher BMI and more frequent comorbidities than the EDA group, but the differences in these and other background factors were not significant.

**Table 1:** Patient backgrounds, clinical data, and perioperative details

		PVB (*N* = 56)	EDA (*N* = 112)	*p*
Age (year)		69 ± 10	69 ± 10	0.828
Sex (M/F)		36/20	72/40	1.000
Performance status (0/>1)		43/13	96/16	0.193
Smoking history (pack-year)		23.4 ± 26.1	28.5 ± 31.4	0.293
BMI (kg/m^2^)		24.2 ± 3.4	22.9 ± 3.1	**0.015**
FEV1.0%		76.1 ± 10.1	74.0 ± 9.6	0.191
Comorbidity	Diabetes Mellitus	10 (17.9%)	10 (8.9%)	0.128
	Acquired heart disease	13 (23.2%)	3 (2.7%)	**<0.001**
	Ischemic cerebrovascular disease	9 (16.1%)	6 (5.4%)	**0.040**
Type of disease	Lung cancer	47	89	0.232
	Metastatic lung tumor	6	21	
	Benign tumor	1	2	
	Emphysema	1	0	
	Mediastinal tumor	1	0	
Type of surgery	Lobectomy	38	76	0.637
	Segmentectomy	10	21	
	Wedge resection	7	15	
	Thymectomy	1	0	
Length of skin incision (cm)		13 ± 2	14 ± 3	0.166
Time length of surgery (min)		210 ± 62	223 ± 77	0.271
Intraoperative blood loss (ml)		65 ± 67	70 ± 65	0.803
Side of surgery (L/R)		26/30	43/69	0.324

*Abbreviations*: *BMI* body mass index, *EDA* epidural anesthesia, *F* female, *FEV1* forced expiratory volume in 1 s, *L*, left, *M* male, *PVB* paravertebral block, *R* right. Data expressed as mean ± standard deviation or n. Bold text indicates a significant value

Comparison of outcomes between the two groups is shown in Table 2. Quantity of fentanyl with ropivacaine administered was significantly larger, but frequency of use of intravenous non-steroidal anti-inflammatory drugs (NSAIDs) smaller, in the PVB group than in the EDA group. Numeric Rating Scale (NRS) pain score on postoperative day 2 did not differ significantly between groups (*p* = 0.334), but the score in the PVB group (3.25 ± 1.80) was not significantly higher than that in the EDA group (3.56 ± 2.05). Moreover, the duration of regional anesthesia required did not differ significantly between the EDA and PVB groups (*p* = 0.477).

**Table 2:** Comparison of analgesia between PVB and EDA groups

	PVB (*N* = 56)	EDA (*N* = 112)	*p*
Duration of regional anesthesia (day)	4.3 ± 0.9	4.4 ± 1.2	0.477
Total consumption of fentanyl (mg)	0.74 ± 0.33	0.36 ± 0.30	<0.001
Total consumption of ropivacaine (g)	20.5 ± 6.6	8.4 ± 21.2	<0.001
NRS at the 2nd postoperative day (0-10)	3.25 ± 1.80	3.56 ± 2.05	0.334
Rescue dose of intravenous NSAIDs	0.7 ± 1.1	1.8 ± 1.8	<0.001

*Abbreviations*: *EDA* epidural anesthesia, *NRS* numeric rating scale, *NSAID* non-steroidal anti-inflammatory drug, *PVB* paravertebral block. Data expressed as mean ± standard deviation

In terms of complications, one patient in the PVB group, an 81-year-old female, suffered from respiratory depression immediately following surgery. This could have been caused by excessive use of ropivacaine; indeed, it resolved quickly after decreasing the rate of infusion of ropivacaine. No complications were observed in any other PVB-group patients. The EDA group displayed no severe complications such as epidural hematoma, post-dural puncture headaches, or spinal cord injury. However, at the end of the 2nd postoperative day, when the urinary catheter placed in the operating room was usually removed, 10 EDA patients (8.9%) had failed a voiding trial, which required reinsertion of the urinary catheter until voiding was successful. No patient in the PVB group had this problem (*p* = 0.03).

## Discussion

The aim of this study was to examine whether thoracic PVB could provide an alternative form of regional anesthesia when used in conjunction with general anesthesia. Our results demonstrate that thoracic PVB was not inferior to EDA in controlling post-thoracotomy pain.

Meta-analyses [15, 16] have shown that PVB provides analgesia comparable to that of EDA and has a better side-effect profile, because it is associated with less postoperative urinary retention, nausea and vomiting, and hypotension. In the present study, no one in the PVB group experienced any of these complications. Reducing the occurrence of these complications, especially in elderly patients, is required for quick postoperative recovery. In addition, Horlocker et al. reported that one of the most devastating complications of epidural anesthesia is spinal cord injury, a rare but catastrophic complication that can result from instrumentation of the epidural space [9]. From an anatomic perspective, PVB has by definition a lower risk of these complications.

While meta-analysis demonstrated a tendency toward less effective pain control with PVB, the difference was not significant. In addition, although some randomized controlled trials showed that PVB was less effective than EDA in controlling postoperative pain [17–19], others showed equivalent pain control in the two groups [4, 20]. The cause or causes of theses discrepancies must be determined, and to do so, we must focus on the technique of catheter insertion.

The meta-analyses included various techniques of catheter insertion, but we believe our current catheter-insertion method of catheter insertion offers better pain control with three reasons. First, this method helps us to avoid massive leakage of local anesthetic from the paravertebral space. Kanazi et al. reported that the failure of pain control using PVB could be attributed to inadequate diffusion of local anesthetic into the paravertebral space [18]. This method enabled us to insert the catheter without the injury of pleura surrounding the paravertebral space; therefore, intraoperative leakage was highly unlikely. Second, this method allows us to start PVB before creating the thoracotomy skin incision. Kotze et al. mentioned in their systemic review that that the group who received PVB before skin incision tended to show better pain control [21], and we also found in our clinical experience that a bolus infusion of local anesthetic before skin incision offered the better pain control. Third, the additional safety of this method is supported by the fact that the paravertebral catheters are placed under direct vision via the thoracoscopy monitor. Although ultrasound-guided catheter insertion has been reported to be safe and reliable [22], continuous PVB may also be problematic in patients with spinal anomalies, trauma, or a history of spine surgery [23]. However, this method makes it easy to confirm visually that the paravertebral space is filled with anesthetic throughout the surgery. Thus, this method of catheter insertion will offer better pain control and safety, so that we suggest that the efficacy of PVB should be reevaluated using this method.

Here, the question may be raised, if PVB is really effective in thoracotomy cases, although more ropivacaine with fentanyl was used in the PVB group than in the EDA group. Messina et al. also observed that a significantly larger amount of local anesthetic and opioid medication was required to achieve the same level of pain control in PVB group [17]. However, this result does not necessarily mean that PVB is less effective than EDA. Because in the present study fentanyl was used intravenously in the PVB group but directly introduced through the epidural catheter in the EDA group, comparison of the two groups from this perspective is difficult. On the other hand, regarding the amount of local anesthesia, we decided to use a higher concentration of local anesthesia when we initiated the use of thoracic PVB at our hospital, because several studies have shown that a greater amount of local anesthetic was required in the PVB group than in the EDA group [4, 8, 24–26]. However, some reports have already demonstrated that higher concentration and rate were not required in PVB groups to achieve the same effect [27–29]. Therefore, the amount of ropivacaine and fentanyl in the PVB group can be reduced to the same level as in the EDA group.

The limitations of this study—sampling bias, selection bias, and recall bias—are usually present in a retrospective case-control study. Regarding sampling bias, there were significant differences in the frequency of three factors in the backgrounds of patients: BMI, the presence of acquired heart disease, and the presence of ischemic cerebrovascular disease. These factors were strongly related to the way in which both groups were chosen, because patients who used anticoagulant or antiplatelet drugs regularly always allocated to the PVB group. As for selection bias, it is difficult to avoid this bias, however, anesthesiologists and surgeons always decided beforehand who would receive thoracic PVB by considering the contraindications for insertion of an epidural catheter. With regard to recall bias, all relevant medical records were used for this retrospective study. However, we acknowledge that, because the completeness of the medical records varied, some patient data were not available.

## Conclusion

In conclusion, thoracic PVB with this thoracoscopic method, which reduced the frequency of urinary retention, was at least as effective as EDA for the postoperative pain control after thoracotomy with lung resection. We believe this procedure can be a good anesthetic alternative, especially for patients with contraindications for EDA.

## Abbreviations

BMI: Body mass index
EDA: Thoracic epidural analgesia
FEV1: Forced expiratory volume in 1 s
NRS: Numeric rating scale
NSAID: Non-steroidal anti-inflammatory drug
PVB: Paravertebral block
VATS: Video-assisted thoracoscopic surgery

## References

[1] Karmakar MK (2001). Thoracic paravertebral block. Anesthesiology.

[2] d’Amours RH, Riegler FX, Little AG (1998). Pathogenesis and management of persistent postthoracotomy pain. Chest Surg Clin N Am.

[3] Marret E, Bazelly B, Taylor G, Lembert N, Deleuze A, Mazoit J-X (2005). Paravertebral block with ropivacaine 0.5% versus systemic analgesia for pain relief after thoracotomy. Ann Thorac Surg.

[4] Richardson J, Sabanathan S, Jones J, Shah RD, Cheema S, Mearns AJ (1999). A prospective, randomized comparison of preoperative and continuous balanced epidural or paravertebral bupivacaine on post-thoracotomy pain, pulmonary function and stress responses. Br J Anaesth.

[5] Gulbahar G, Kocer B, Muratli SN, Yildirim E, Gulbahar O, Dural K (2010). A comparison of epidural and paravertebral catheterisation techniques in post-thoracotomy pain management. Eur J Cardio-thoracic Surg.

[6] Richardson J, Lönnqvist PA (1998). Thoracic paravertebral block. Br J Anaesth.

[7] Bimston DN, McGee JP, Liptay MJ, Fry WA (1999). Continuous paravertebral extrapleural infusion for post-thoracotomy pain management. Surgery.

[8] Luketich JD, Land SR, Sullivan E, Alvelo-Rivera M, Ward J, Buenaventura PO (2005). Thoracic epidural versus intercostal nerve catheter plus patient-controlled analgesia: A randomized study. Ann Thorac Surg.

[9] Horlocker TT, Abel MD, Messick JM, Schroeder DR. Small risk of serious neurologic complications related to lumbar epidural catheter placement in anesthetized patients. Anesth. Analg. 2003;96:1547-1552. 10.1213/01.ANE.0000057600.31380.75 12760972

[10] Berrisford RG, Sabanathan SS (1990). Direct access to the paravertebral space at thoracotomy. Ann Thorac Surg..

[11] Fibla JJ, Molins L, Mier JM, Sierra A, Carranza D, Vidal G (2011). The efficacy of paravertebral block using a catheter technique for postoperative analgesia in thoracoscopic surgery: a randomized trial. Eur J Cardiothorac Surg..

[12] Elsayed H (2012). Insertion of paravertebral block catheters intraoperatively to reduce incidence of block failure. Interact Cardiovasc Thorac Surg..

[13] Soni AK, Conacher ID, Waller DA, Hilton CJ (1994). Video-assisted thoracoscopic placement of paravertebral catheters: a technique for postoperative analgesia for bilateral thoracoscopic surgery. Br J Anaesth..

[14] Feltracco P, Ori C (2007). A new look at the paravertebral block: a percutaneous video-assisted technique. Reg Anesth Pain Med..

[15] Ding X, Jin S, Niu X, Ren H, Fu S, Li Q (2014). A comparison of the analgesia efficacy and side effects of paravertebral compared with epidural blockade for thoracotomy: an updated meta-analysis. PLoS One.

[16] Davies RG, Myles PS, Graham JM (2006). A comparison of the analgesic efficacy and side-effects of paravertebral vs epidural blockade for thoracotomy--a systematic review and meta-analysis of randomized trials. Br J Anaesth.

[17] Messina M, Boroli F, Landoni G, Bignami E, Dedola E, N’zepa Batonga J (2009). A comparison of epidural vs. paravertebral blockade in thoracic surgery. Minerva Anestesiol.

[18] Kanazi GE, Ayoub CM, Aouad M, Abdallah F, Sfeir PM, Adham A-BF (2012). Subpleural block is less effective than thoracic epidural analgesia for post-thoracotomy pain: a randomised controlled study. Eur J Anaesthesiol.

[19] Helms O, Mariano J, Hentz J-G, Santelmo N, Falcoz P-E, Massard G (2011). Intra-operative paravertebral block for postoperative analgesia in thoracotomy patients: a randomized, double-blind, placebo-controlled study. Eur J Cardiothorac Surg.

[20] Pintaric TS, Potocnik I, Hadzic A, Stupnik T, Pintaric M, Novak Jankovic V (2011). Comparison of continuous thoracic epidural with paravertebral block on perioperative analgesia and hemodynamic stability in patients having open lung surgery. Reg Anesth Pain Med..

[21] Kotzé A, Scally A, Howell S (2009). Efficacy and safety of different techniques of paravertebral block for analgesia after thoracotomy: a systematic review and metaregression. Br J Anaesth.

[22] Shibata Y, Nishiwaki K (2009). Ultrasound-guided intercostal approach to thoracic paravertebral block. Anesth Analg.

[23] Burns DA, Ben-David B, Chelly JE, Greensmith JE (2008). Intercostally placed paravertebral catheterization: An alternative approach to continuous paravertebral blockade. Anesth Analg.

[24] Kaiser AM, Zollinger A, De Lorenzi D, Largiadèr F, Weder W (1998). Prospective, randomized comparison of extrapleural versus epidural analgesia for postthoracotomy pain. Ann Thorac Surg.

[25] Dhole S, Mehta Y, Saxena H, Juneja R, Trehan N (2001). Comparison of continuous thoracic epidural and paravertebral blocks for postoperative analgesia after minimally invasive direct coronary artery bypass surgery. J Cardiothorac Vasc Anesth.

[26] Elsayed H, McKevith J, McShane J, Scawn N (2012). Thoracic epidural or paravertebral catheter for analgesia after lung resection: is the outcome different?. J Cardiothorac Vasc Anesth.

[27] Kobayashi R, Mori S, Wakai K, Fukumoto K, Saito T, Katayama T (2013). Paravertebral block via the surgical field versus epidural block for patients undergoing thoracotomy: a randomized clinical trial. Surg Today.

[28] Okajima H, Tanaka O, Ushio M, Higuchi Y, Nagai Y, Iijima K (2015). Ultrasound-guided continuous thoracic paravertebral block provides comparable analgesia and fewer episodes of hypotension than continuous epidural block after lung surgery. J Anesth.

[29] Yoshida T, Fujiwara T, Furutani K, Ohashi N, Baba H (2014). Effects of ropivacaine concentration on the spread of sensory block produced by continuous thoracic paravertebral block: A prospective, randomised, controlled, double-blind study. Anaesthesia.

